# From Hydrophilic to Superhydrophobic: Tuning Surface Wettability through Salvinia-Inspired Topographies

**DOI:** 10.1021/acsami.5c07461

**Published:** 2025-07-25

**Authors:** Kai Liu, Marco Sorgato, Enrico Savio

**Affiliations:** Department of Industrial Engineering, 165501 University of Padua, Via Venezia 1, Padova 35131, Italy

**Keywords:** surface functionalization, superhydrophobic, surface design, 3D structures, bioinspired, two photon polymerization

## Abstract

The development of superhydrophobic surfaces traditionally relies on combining surface roughness with low-surface-energy coatings. In contrast, this work demonstrates the use of two-photon polymerization to induce superhydrophobicity on hydrophilic substrates solely through structural design. A comprehensive set of Salvinia-inspired microstructures was fabricated with precise control over geometrical features such as the number of arms, arm diameter, fill configuration, spacing, and height. Static contact angle measurements revealed that surface architecture plays a pivotal role in modulating wettability, with optimized structures achieving contact angles above 160° without any chemical modification. The study further investigates how morphological fidelity, governed by two-photon polymerization (TPP) printing parameters—specifically slicing distance and hatching distance—influences surface quality, roughness, and droplet behavior. Power spectral density analysis and 3D surface topography confirm that fabrication resolution critically impacts the performance of designed features. Finally, fabrication efficiency was evaluated in terms of areal fabrication rate, highlighting trade-offs among design complexity, printing resolution, and throughput. The results establish a set of design principles for achieving superhydrophobicity on hydrophilic materials and provide a scalable framework for future applications in microfluidics, biomimetics, and surface engineering where chemical-free wettability control is desired.

## Graphical abstract

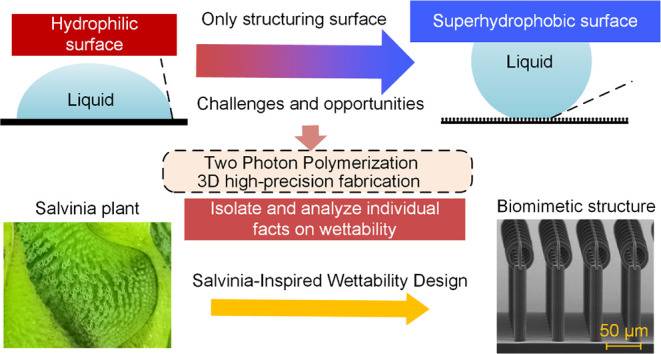

## 1 Introduction

Due to their unique properties, including self-cleaning,^1^ oil–water separation,^2–4^ antifogging,^5^ and antifouling behavior,^6^ superhydrophobic surfaces (SHS) have attracted significant scientific and industrial interest. Typically, SHS can be realized either by chemically modifying low-surface-energy materials or by engineering hierarchical surface topographies at the micro- and nanoscale, often inspired by biological structures such as lotus leaves or Salvinia.^7,8^

When dealing with hydrophilic materials, achieving superhydrophobicity typically requires a combination of surface structuring and chemical treatment. While fluorinated coatings and other low-surface-energy compounds are effective, they raise concerns about long-term durability,^9^ environmental impact,^10^ chemical degradation,^11^ and potential toxicity.^12^ In contrast, geometric approaches that utilize micro- and nanoscale surface structuring can enhance air entrapment and surface repellency without chemical modifications. These methods offer improved mechanical robustness, biocompatibility, and compatibility with a wide range of substrates.

Several advanced fabrication techniques for producing structured surfaces have been explored, including cutting, abrasive machining, beam-based methods, electrochemical machining, and chemically assisted manufacturing. Although each has unique advantages, these methods often struggle to meet the resolution, complexity, and flexibility required for the reliable production of functional superhydrophobic surfaces.^13^ Additive manufacturing (AM) techniques have played a key role in the fabrication of functional surfaces.^14^ For example, 3D-printed multireaction platforms enable the modular integration of multiple sequential chemical reactions;^15^ tunable wettability gradients—achieved by adjusting printing parameters—allow precise control over liquid movement;^4^ micro/nano-porous and salvinia architectures produced via 3D printing achieve efficient oil–water separation;^2,3^ and combined printing and postprocessing methods yield surfaces with underwater superoleophobic performance.^16^ Two-photon polymerization (TPP) has emerged as a highly promising technique in this context. It enables the direct fabrication of intricate three-dimensional structures with submicrometer resolution, thus overcoming the design limitations of traditional microfabrication processes.^17^

Recent studies have increasingly turned to TPP to investigate the relationship between surface architecture and wettability. Xiang et al.^18^ utilized SU-8, an intrinsically hydrophobic photoresist, to create Salvinia-inspired structures capable of sustaining a wet-slip air cushion underwater by capturing and retaining air within the surface features. Liimatainen et al.^19^ designed a biomimetic double reentrant geometry in IP-S, which was subsequently replicated in PDMS. This replication allowed for a controlled transition from hydrophobic to superhydrophobic behavior, underscoring the interaction between material chemistry and structural design.

Lin et al.^20^ constructed deterministic hierarchical geometries, such as Sierpinski tetrahedrons and pyramidal structures, which slightly improved wettability (up to 102°) but offered higher fabrication efficiency. In parallel, IP-Dip—a weakly hydrophilic resin—was patterned into cubic, pyramidal, and tetrahedral motifs, increasing contact angles from 80.5° (unstructured) to 91.9°, 108.5°, and 130.2°, respectively. When coated with HMDSO, the surface became superhydrophobic, reaching a contact angle of 173.9°.^21^ Similarly, lotus-inspired structures fabricated in IP-Dip achieved angles up to 119°,^22^ while Salvinia-inspired microstructures created with IP-DILL reached 122° but did not reach the superhydrophobic regime.^23^

These findings underscore the significant influence of geometrical design and substrate chemistry on achieving superhydrophobicity. While combining TPP with traditional microfabrication processes (e.g., dip coating or microcontact printing) has proven effective in replicating Salvinia-like surfaces,^24^ such hybrid approaches add complexity to the fabrication workflow and reduce scalability. Most prior studies rely on inherently hydrophobic materials or post-treatment processes, revealing a persistent challenge: achieving superhydrophobicity on hydrophilic substrates solely through structural design.

TPP’s ability to fabricate complex 3D architectures at submicrometer resolution makes it an ideal platform for isolating and analyzing the influence of individual geometric parameters on surface wettability. In this work, a comprehensive investigation is conducted into the design and fabrication of controlled micro- and nanoscale surface structures inspired by the Salvinia effect to achieve superhydrophobicity on hydrophilic substrates exclusively through two-photon polymerization without the use of chemical modifications.

The design evolution from basic 2D and 3D motifs to advanced Salvinia-like architectures is examined, focusing on the influence of key geometrical parameters—including arm configuration (number, diameter, shape, and fill), structure height, and spacing—on the resulting wettability. Additionally, the effects of arm symmetry, printing parameters, and structural fidelity are evaluated in relation to surface performance. The relationship between design complexity and fabrication efficiency is further characterized by analyzing the areal fabrication rate (AFR).

This study offers novel insights into geometry-governed wettability modulation and contributes to developing scalable strategies for engineering superhydrophobic surfaces on hydrophilic materials.

## 2 Material and Methods

### 2.1 Surface Design

The design of superhydrophobic surfaces on intrinsically hydrophilic materials requires a deliberate geometrical strategy informed by wetting theory and inspired by biological systems. Wettability on structured surfaces is typically described using two classical models: the Wenzel model^25^ and the Cassie–Baxter model.^26^ Each provides insight into the interaction between liquid droplets and microstructured topographies.

The Wenzel model assumes homogeneous wetting of a rough surface, where the liquid fully penetrates the surface features. The apparent contact angle *θ_W_* on a rough surface is related to the intrinsic Young’s contact angle *θ_Y_* by

1 $$\cos\theta_{W}=r\;\cos\theta_{Y}$$

where *r* is the roughness factor, defined as the ratio of the actual surface area to its projected area. For hydrophilic materials (*θ*_*Y*_ < 90°), increasing surface roughness (*r* > 1) leads to a decrease in *θ_W_*, thereby enhancing wetting. The routine structuring of hydrophilic surfaces to increase roughness only makes them more hydrophilic.^27^ This behavior presents a challenge when attempting to induce superhydrophobicity on such substrates using surface structuring alone.

Alternatively, the Cassie–Baxter model describes a heterogeneous wetting state, where the droplet partially rests on solid and partially on air trapped in surface cavities. The apparent contact angle *θ*_*CB*_ in this state is given by

2 $$\cos\theta_{CB}=f_{s}\cos\theta_{Y}+f_{v}\cos\theta_{v}$$

,where *f_s_* and *f_v_* are the fractions of the solid–liquid and air–liquid contact areas, respectively, and *θ*_*v*_ ∼ 180° for the air interface. This configuration significantly increases the apparent contact angle. However, it is difficult to maintain a stable Cassie–Baxter state on hydrophilic materials because the liquid tends to infiltrate the microstructures, resulting in a mixed Cassie–Baxter/Wenzel (CB–W) regime.

A natural example of such hybrid wetting behavior is found in the Salvinia leaf, which combines hydrophilic anchor points with surrounding hydrophobic structures to retain air layers underwater over extended periods.^28^ This unique functionality provides a design paradigm for synthetic superhydrophobic surfaces.

In this study, Salvinia-inspired structures are designed and fabricated using two-photon polymerization (TPP) to investigate the ability to achieve superhydrophobicity on hydrophilic materials through geometry alone. The structures transition from simple 2D pillar arrays to 3D configurations consisting of a central pillar surrounded by radially distributed arms. These geometries are intended to maximize air trapping beneath the droplet while minimizing the actual contact area with the solid, thus promoting a quasi-Cassie regime.

Key geometrical parameters considered in the design include pillar diameter and height, arm number, diameter, shape (square, rounded, circular), filling (hollow, partially filled, solid), and interpillar spacing. These parameters were selected for their theoretical influence on *f_s_*, *f_v_*, and overall roughness *r*, are thus critical in steering the surface toward a superhydrophobic state despite the hydrophilic nature of the base material.

The biological inspiration and engineered implementation are illustrated in Figure 1a–c and 1 d–e, respectively, showing the transition from natural morphology to fabricated microstructures. The complete design space explored in this work, including all tested values for each parameter, is summarized in Table 1. This parametric framework forms the foundation for analyzing how geometric configurations influence wettability, contact angle transitions, and fabrication efficiency. Moreover, by enlarging the arm-ring diameter, the evolution of surface architectures from conventional nonsuperhydrophobic pillar arrays to Salvinia-inspired structures is elucidated; further increasing the number of arms allows the investigation of the transition from superhydrophobic Salvinia structures to three-dimensional conventional nonsuperhydrophobic architectures composed of pillars and spheres. The structural parameters and their identifiers are summarized in Table 2. The arm diameter is fixed in 5 μm. The advanced wettability test parameters—advancing contact angle (ACA), receding contact angle (RCA), contact angle hysteresis (CAH), and droplet-evaporation—were taken from the entries in Table 1 for the present experiments. The corresponding surface geometries are summarized in Table 3.

**Figure 1:**
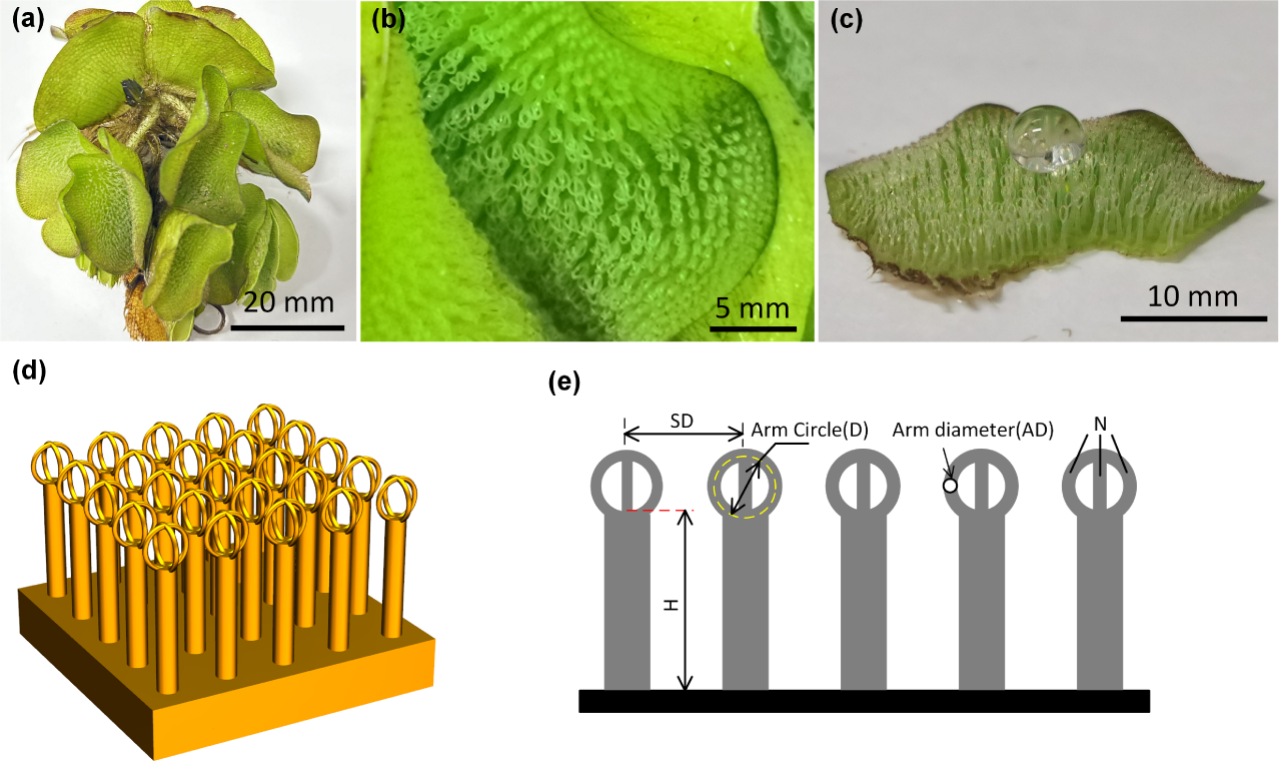
(a) The overall Salvinia plant; (b) the microscopic structure of the Salvinia leaf, showing the ring-shaped hairs on the surface; (c) the Salvinia leaf exhibits superhydrophobicity with a water droplet; (d) a 3D schematic representation; (e) a 2D cross-sectional schematic with key geometric parameters labeled.

**Table 1:** Geometrical Parameters Used in the Design of Salvinia-Inspired Microstructured Surfaces^a^

Parameter	Value
Pillar diameter (μm)	20 μm
Spacing distance (μm)	40, 50, 60, 70, 80, 90, 120, 150, 180, 210, 240, 270, 300, 330, 360 μm
Height (μm)	0, 60, 120 μm
Arm circle (μm)	20, 25, 30, 40 μm
Arm number	2, 3, 4, 5, 6, 7, 8, 9, 10, 11, 12, 13, 15, 20, 25
Arm diameter (μm)	2.5, 5, 7.5, 10 μm
Ball diameter (μm)	20, 30, 40, 45 μm
Arm shape	Square, R1, R2, circle
Arm filling	Hollow, half-hollow, half-radius, solid

a The values were systematically varied to investigate the influence of each parameter on wettability behavior, air retention capability, and fabrication efficiency.

**Table 2:** Geometrical Parameters and Sample Identifiers for Microstructured Surfaces with Varied Arm-Ring Diameters and Arm Numbers, Used to Investigate the Transition from Conventional Non-Superhydrophobic Pillar Arrays to Salvinia-Inspired Textures and Three-Dimensional Pillar Sphere Architectures^a^

Number	Type	Parameter	Value
1	Flat	/	/
2	Pillar	Pillar diameter (μm)	20
		Spacing distance (μm)	60
		Height (μm)	120
3–6	Salvinia-inspired structure	Arm circle (μm)	20, 25, 30, 40
6–20		Arm number	2, 3, 4, 5, 6, 7, 8, 9, 10, 11, 12, 13, 15, 20, 25
21–24	Convention 3D structure	Ball diameter (μm)	45, 40, 30, 20

a Underlined parameters indicate invariants.

**Table 3:** Geometric Parameters for Advanced Wettability Parameters—Advancing Contact Angle (ACA), Receding Contact Angle (RCA), Contact Angle Hysteresis (CAH), and Droplet-Evaporation^a^ ^b^

Parameter	Value
Spacing distance (μm)	60, 80 μm
Arm number	2, 3, 6
Arm diameter (μm)	2.5, 5
Pillar diameter (μm)	20 μm
Height (μm)	120 μm
Arm circle (μm)	40 μm

a Each sample label in the figures concatenates the abbreviations of the varying geometric parameters with their respective numerical values.

b (e.g., N2-SD60-AD5, where *N* = arm number, SD = spacing distance in μm, and AD = arm diameter in μm).

### 2.2 Fabrication of Micro/Nanostructured Surfaces via TPP

Micro- and nanostructured surfaces were fabricated using two-photon polymerization (TPP), a laser-based additive manufacturing technique capable of producing submicrometer features with high spatial resolution and design flexibility.^29,30^ The 3D structural models were generated in CAD software and exported in STL format. These were processed using DeScribe (Nanoscribe GmbH), which converted them into printable instructions by defining the slicing and hatching strategy. Printing parameters such as slicing distance (SLD), hatching distance (HD), and laser power were configured based on the desired resolution and structural complexity. The printing process was performed using a Nanoscribe Photonic Professional GT system equipped with a femtosecond pulsed laser (maximum output: 50 mW). Owing to the nonlinear absorption mechanism intrinsic to TPP, polymerization occurs only at the laser focus, where two photons are absorbed simultaneously. This confined reaction enables voxel-scale resolution and high spatial selectivity, while the surrounding resin remains uncured. A schematic overview of the fabrication workflow, including laser focusing, exposure, and postprocessing, is presented in Figure 2.

**Figure 2:**
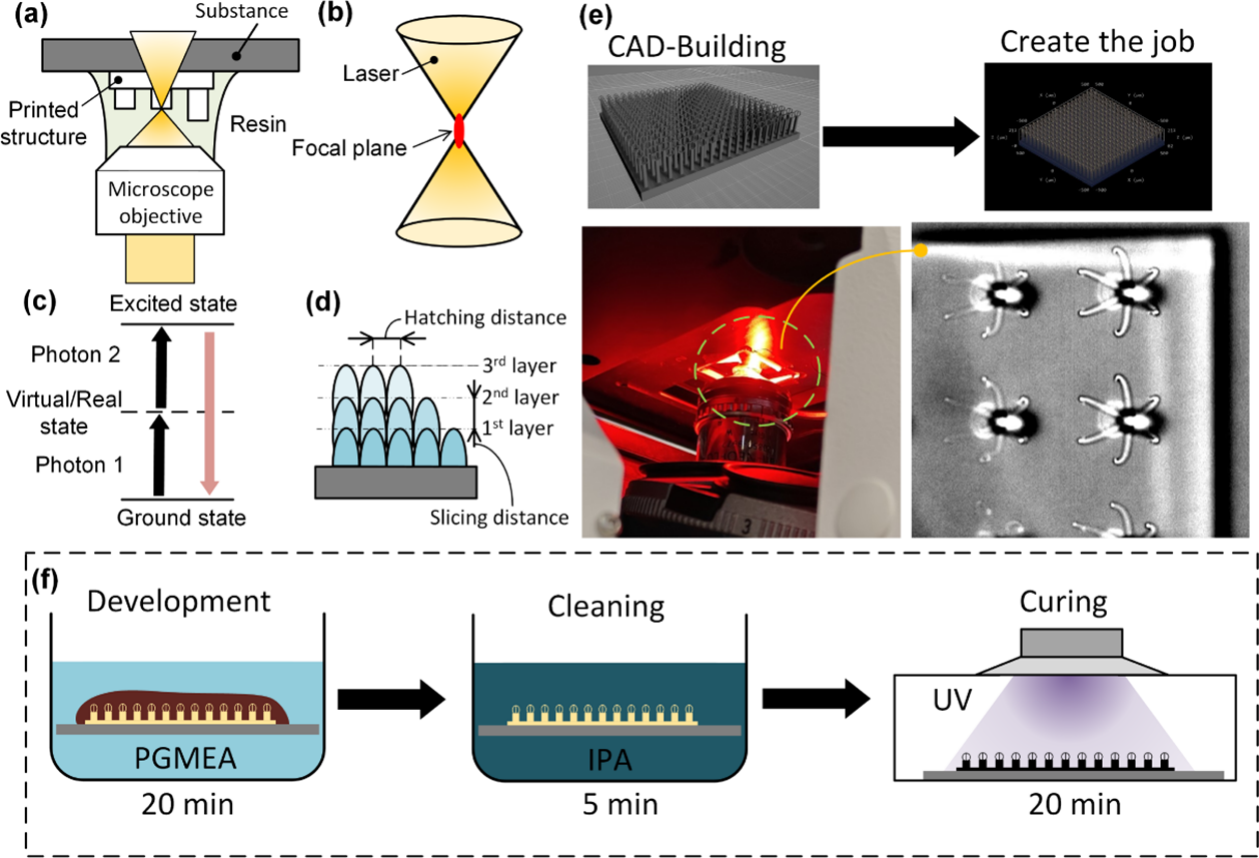
Schematic representation of the fabrication process based on two-photon polymerization (TPP). (a) Optical setup of the TPP system employing a femtosecond laser for localized polymerization. (b) Focusing of the laser beam into the photoresist, where polymerization occurs via nonlinear absorption. (c) Energy level diagram illustrating the principle of two-photon absorption. (d) Layer-by-layer printing strategy, with defined slicing and hatching distances. (e) CAD model preparation and transfer to the TPP system for fabrication. (f) Postprocessing steps include development in PGMEA, rinsing in IPA, and optional UV postcuring to ensure complete cross-linking and surface uniformity.

All structures were fabricated using IP-S photoresist (Nanoscribe GmbH), selected for its high print fidelity, mechanical strength, and compatibility with submicron features. The resin was deposited on 25 × 25 × 0.7 mm^3^ glass substrates coated with a single-sided indium tin oxide (ITO) layer with a thickness of 18 ± 5 nm and a sheet resistance of 100–300 Ω. The ultrathin ITO affords a weakly reflective yet laser-transparent surface for precise autofocus during two-photon writing, while the underlying glass provides a dimensionally stable, optically clear platform for high-resolution fabrication and subsequent characterization of the microstructures. The chemical, mechanical, and physical properties of the IP-S photoresist are summarized in Table 4, as they influence polymerization kinetics, resolution, and the resulting surface behavior.

**Table 4:** General and Physical Properties of the IP-S Photoresist Used in Two-Photon Polymerization

Property	Value
Reactive group	Methacrylate
Curing mechanism	Free radical polymerization (FRP)
Wettability	Hydrophobic/resin hydrophilic/flat cured structures
Indentation modulus	5.11 GPa
Vickers hardness	20.68 HV0.0025
Indentation hardness	223.33 MPa
Storage modulus	5.33 GPa
Loss modulus	0.26 GPa
Density (liquid)/20 °C	1.111 g/cm^3^
Density (solid)/20 °C	1.19 g/cm^3^@20 °C
Shrinkage after polymerization	2–12%

The laser writing followed a layer-by-layer approach, with specific slicing and hatching distances adjusted depending on the complexity and resolution needed. The values adopted for these parameters, along with laser power, are listed in Table 5. Following laser exposure, the samples underwent a standard development process involving immersion in propylene glycol monomethyl ether acetate (PGMEA) for 20 min to remove uncured resin, followed by a 5 min rinse in isopropanol (IPA). Although UV postcuring is not strictly required for structures fabricated via TPP, all samples were exposed to UV light for 20 min to ensure complete polymer cross-linking. This step helped eliminate variability in surface chemistry that could otherwise affect wettability measurements.

**Table 5:** Process Parameters Used for the Fabrication of Microstructured Surfaces via Two-Photon Polymerization

Parameter	Value
Slicing distance (SLD)	1 μm
Hatching distance (HD)	0.5 μm
Laser Power	50 mW

The areal fabrication rate (AFR) was calculated using the eq 3

3 $$\mathrm{AFR}=\frac{A}{T}\;\left({\mathrm{mm}}^{2}/h\right)$$

where *A* is the total printed area (in square millimeters) and *T* is the total fabrication time (in hours). This parameter was used to assess process efficiency across different design configurations and printing conditions. The methodology described above ensured consistent fabrication quality and provided a reliable basis for correlating structural design with surface functionality.

### 2.3 Surface and Functional Characterization

To evaluate the morphological accuracy and surface quality of the fabricated microstructures, surface characterization was performed using both optical profilometry and scanning electron microscopy (SEM). The topography and roughness were assessed with a Sensofar Neox profilometer equipped with a 150× objective in confocal mode. Data were processed using MountainsLab and SensoView software packages.

Dimensional spacing was defined as the lateral distance between the highest points of adjacent structures, while the structural height was calculated as the vertical distance from the peak of the structure to the baseline, subtracting the theoretical nonpillar height. Each value was determined by averaging six measurements to ensure statistical consistency.

For detailed morphological analysis, 3D surface topographies were extracted by isolating the upper region of the structures. For visualization and validation, SEM imaging was conducted using an FEI Quanta 450 microscope. Prior to imaging, the samples were sputter-coated with a conductive layer using a 20 mA current for 240 s to minimize charging effects and improve contrast.

Static contact angle (CA) measurements were performed to characterize the surfaces’ functional performance and assess their wettability. Wettability was classified into four categories: superhydrophilic (0°–10°), hydrophilic (10°–90°), hydrophobic (90°–150°), and superhydrophobic (150°–180°). Although the boundaries between categories may vary in the literature, these thresholds were adopted to ensure consistent interpretation across experimental conditions.^31^

Measurements were conducted using a custom goniometer setup, consisting of a precision-controlled glass syringe (100 μL) mounted on a syringe pump, a five-axis adjustable sample platform, and a back-illuminated optical imaging system with a microlens and Cold-LED blue light source.^32^ A schematic of the complete goniometer setup is shown in Figure 3. Water droplets (1 μL in volume) were dispensed at a flow rate of 5 μL/min using deionized water (34877, Sigma-Aldrich) stored in a clean glass container. Droplet profiles were captured and processed using dedicated software connected to the imaging system. Each contact angle value was obtained by averaging five independent measurements per sample to ensure repeatability and reduce random errors. The procedure for measuring the advancing and receding contact angles is as follows: A 1 μL droplet is first dispensed to contact the surface. To minimize the influence of dynamic effects on the contact angle variation, the injection speed is reduced to 1 μL/min. A total of 5 μL of liquid is then slowly injected and subsequently withdrawn at the same rate. Images are captured at 100 ms intervals, and the contact angles are determined based on the recorded images. Due to the excessive superhydrophobicity of the surface, a larger volume of liquid and a stronger gravitational force are required for the droplet to detach from the needle stably. Therefore, the initial liquid volume was set to 8 μL, the ambient temperature was maintained at 20 ± 2 °C, and the acquisition rate was 1 frame every 2 s. The critical Laplace pressure is calculated as

4 $$P_{\mathrm{crit}}=\frac{2\gamma }{R}$$

where γ is the liquid–gas surface tension (for water at room temperature, γ ≈ 0.072 N/m), and *R* is the fluid–air interface’s radius of curvature at the wetting transition.

**Figure 3:**
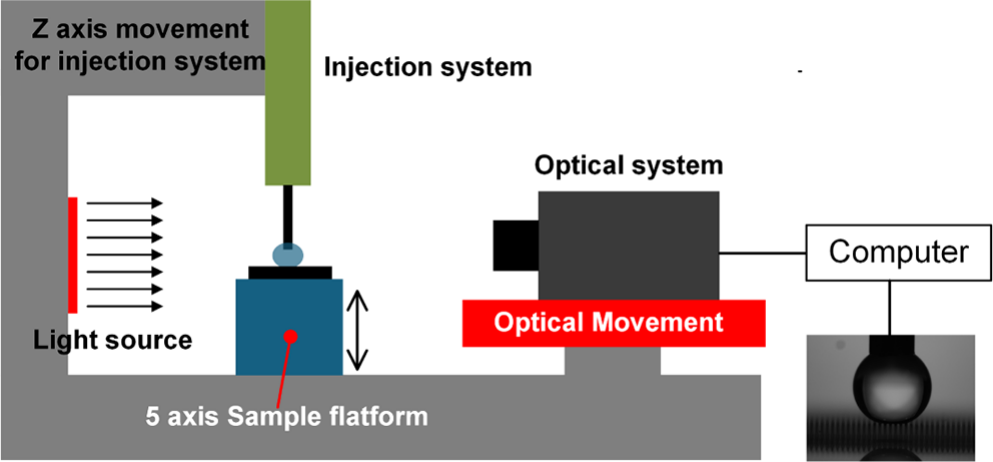
Schematic representation of the custom-built goniometer setup used for static contact angle measurements. The system includes a glass syringe mounted on a syringe pump for controlled droplet deposition, a five-axis sample positioning stage for precise alignment, and an optical module with microlens and Cold-LED blue backlight for enhanced droplet profile imaging.

## 3 Results

### 3.1 Effect of Surface Architecture on Wettability

This section presents a systematic investigation of how architectural complexity influences surface wettability. Using two-photon polymerization, a series of microstructured surfaces with progressively increasing geometrical intricacy was fabricated, enabling precise control over design parameters and high-resolution morphological fidelity. Starting from a flat surface as a reference, increasingly complex configurations were generated by adding cylindrical pillars, modifying their upper terminations, and integrating Salvinia-inspired arm-like extensions. The experimental goal was to disentangle the effects of individual topographic features—such as curvature, arm number, radial arrangement, and structural closure—on the static contact angle while leveraging TPP as a versatile prototyping tool for functional surface analysis.

Figure 4 illustrates the relationship between surface complexity and contact angle, combining both quantitative measurements and qualitative visualizations of the droplet profile and microstructure morphology. Graph (a) reports the static contact angle as a function of surface complexity, while panel (b) provides corresponding SEM images and side-view droplet photographs for each configuration.

**Figure 4:**
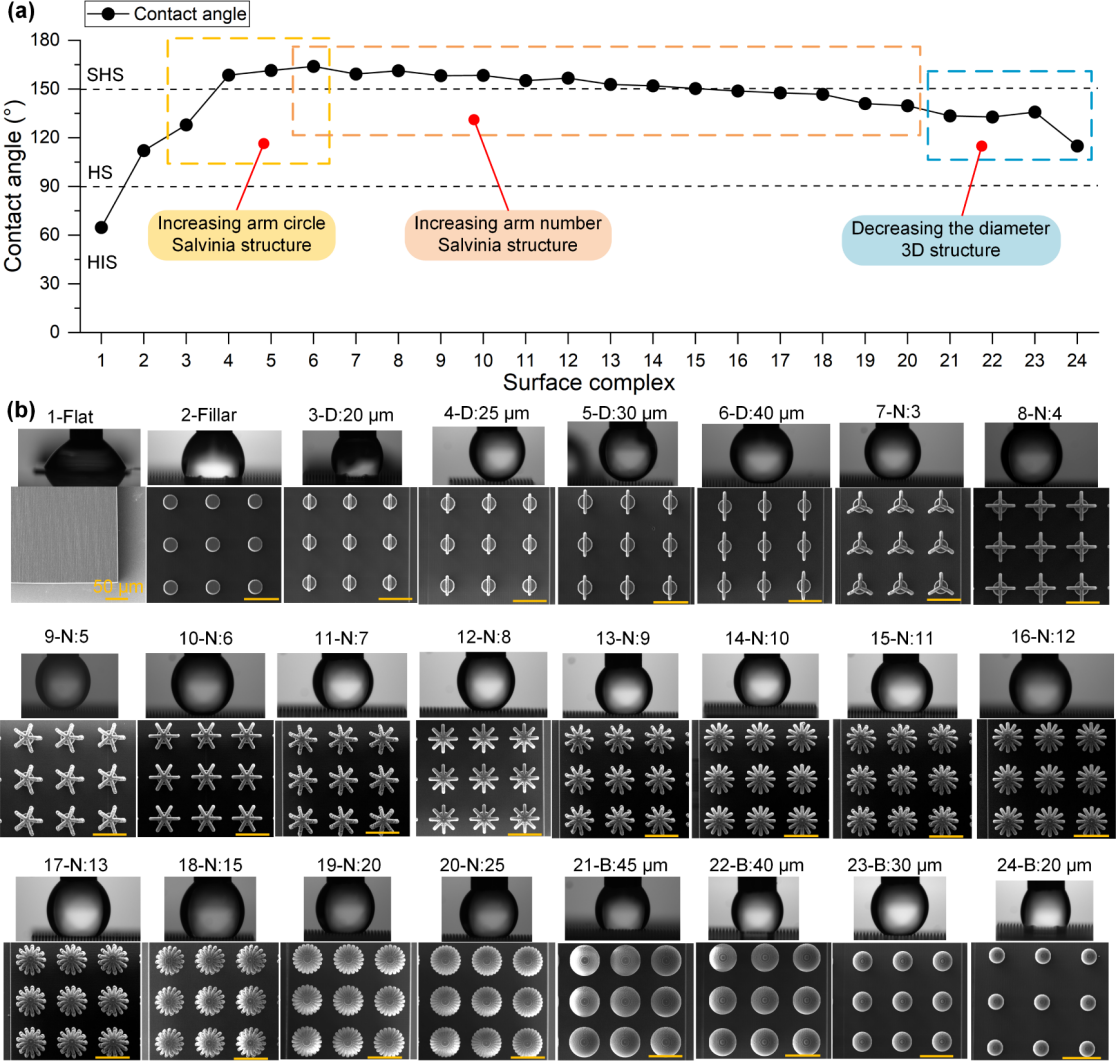
(a) Static contact angle as a function of surface architecture complexity, progressing from flat surfaces to pillar arrays, 3D spherical cap structures, Salvinia-inspired designs with varying numbers of arms, and spherical shell-like enclosures. (b) SEM and side-view optical images corresponding to selected surface configurations.

The planar surface (sample 1-Flat) was used as a baseline reference, displaying complete wetting behavior. Introducing periodic cylindrical pillars (sample 2-Pillar) immediately shifted the wetting regime to a hydrophobic state, yielding a contact angle of 112°, though still below the superhydrophobic threshold. This enhancement was attributed to the topographic roughness created by the pillars, which favored partial air retention and reduced contact area between the liquid and the substrate.

To further refine the design, two radially opposed arms were introduced atop the vertical pillars, forming simplified 3D configurations with increasing circular spans (samples 3-D to 6-D). These structures emulate basic transitions toward Salvinia-like geometries by incrementally enlarging the diameter of the circular arc defined by the two arms. As shown in Figure 4a, the contact angle increased with arm diameter, reaching a maximum of 164° in sample 6-D with a 40 μm arc. This configuration exhibited the highest droplet repellency due to maximized air retention and minimized liquid penetration into the interarm cavity. These results demonstrate that even basic geometrical refinements—such as widening the arc formed by opposing arms—can significantly influence wettability. However, full superhydrophobicity remained challenging to achieve without further architectural elaboration.

Further variations were introduced by increasing the number of arms from 3 to 25 (samples 7-N to 20-N). Interestingly, despite expectations that more arms would continue to increase air trapping, the contact angle gradually decreased as the number of arms grew beyond a certain threshold. This counterintuitive behavior suggests that excessive branching may lead to structural collapse or facilitate water infiltration, reducing the efficiency of the composite wetting state.

To explore the transition from branched to fully enclosed geometries, samples 21-B to 24-B were fabricated, in which the arms collapsed into continuous spherical shells centered on the pillar. These spherical structures represent a morphological end point of the arm extension process, forming domes of increasing curvature with decreasing diameter. Sample 21-B featured the widest spherical cap with a 45 μm diameter, while sample 24-B had the narrowest at 20 μm. This transition enabled the investigation of how enclosed curvature influences droplet behavior. Although these structures maintained a high level of symmetry and continuity, they also reduced structural sharpness and air pocket segmentation, which are key to sustaining the Cassie–Baxter regime. As a result, the contact angle decreased across this series, confirming that while enclosing geometries can support droplet suspension, they may also limit superhydrophobic performance if not sufficiently open or segmented.

In our study, the maximum static contact angle occurred with the two-arm configuration, while more arms reduced hydrophobic performance. This indicates a nonmonotonic relationship between feature density and wetting. Although two-arm structures were not optimized for spacing, height, and diameter, an optimized design could enhance superhydrophobicity, suggesting a worthwhile avenue for future research.

To investigate the impact of structural details further, the effect of arm diameter was studied independently, using configurations with fixed arm number and shape. Figure 5 shows the variation of contact angle as a function of arm diameter for two representative structures: one with three arms and one with six arms. As the arm diameter increased from 2.5 to 10 μm, a consistent decline in contact angle was observed in both cases. Specifically, in the three-armed configuration, the contact angle dropped from 165° to 155.8°, while in the six-armed configuration it decreased from 163.4° to 151.2°. This trend suggests that increasing the arm diameter reduces the void volume between the arms and increases the effective solid–liquid contact area, thereby limiting air entrapment and diminishing superhydrophobic performance.

**Figure 5:**
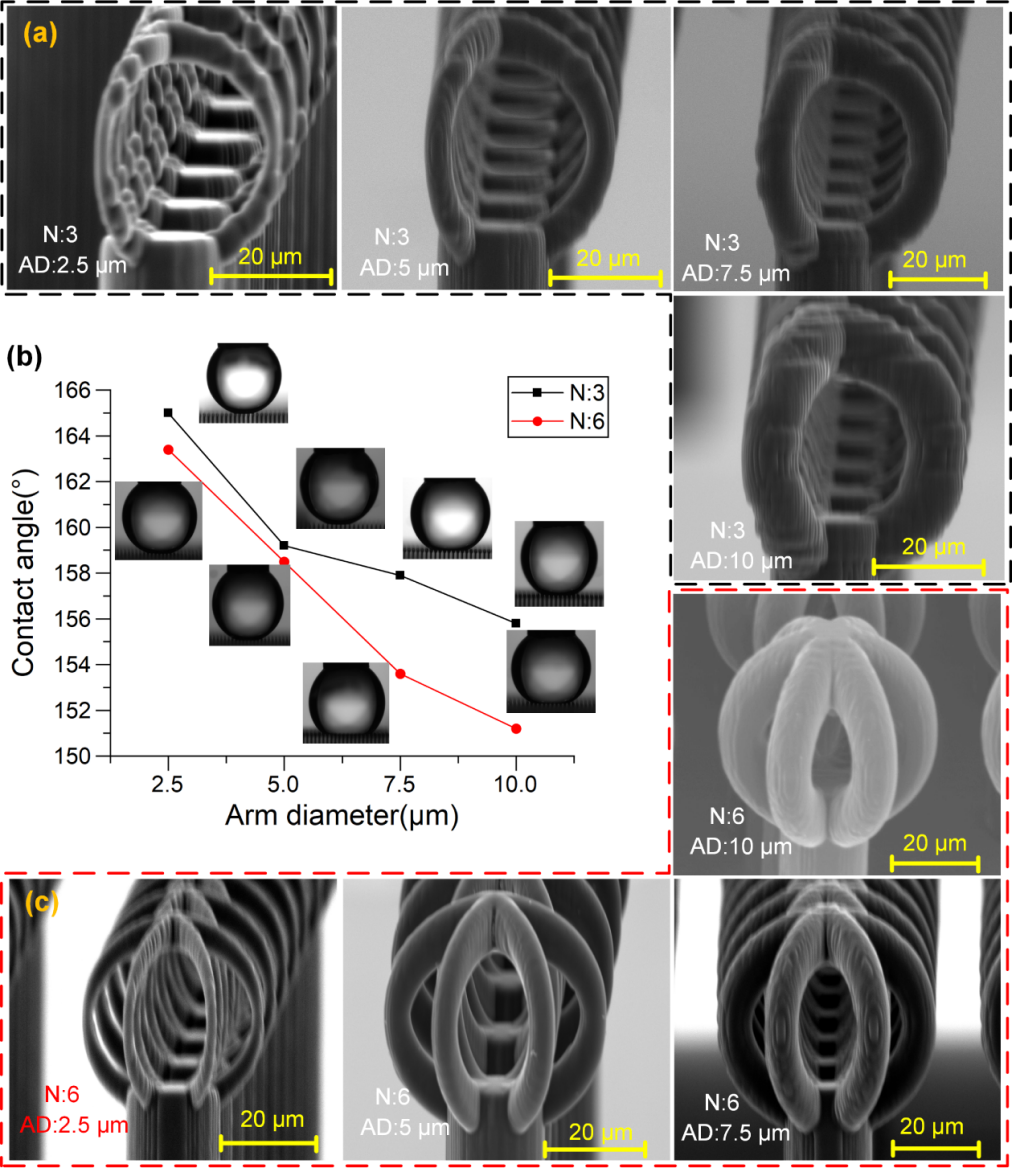
(a) SEM images of Salvinia-inspired structures with three arms (*N* = 3) and increasing arm diameters (AD = 2.5 μm, 5 μm, 7.5 μm, and 10 μm). (b) Static contact angle measurements as a function of arm diameter for configurations with three arms (black squares) and six arms (red circles). (c) SEM images of Salvinia-inspired structures with three arms (*N* = 6) and increasing arm diameters (AD = 2.5 μm, 5 μm, 7.5 μm, and 10 μm).

Figure 6 summarizes the influence of arm shape. When the cross-sectional profile of the arms was modified from circular to square by reducing the fillet radius, a slight increase in contact angle was observed. The circular section yielded a CA of 164°, whereas the square section produced 165.1°. Although marginal, this enhancement may stem from sharper geometrical features promoting localized air entrapment at the triple-phase boundary.

**Figure 6:**
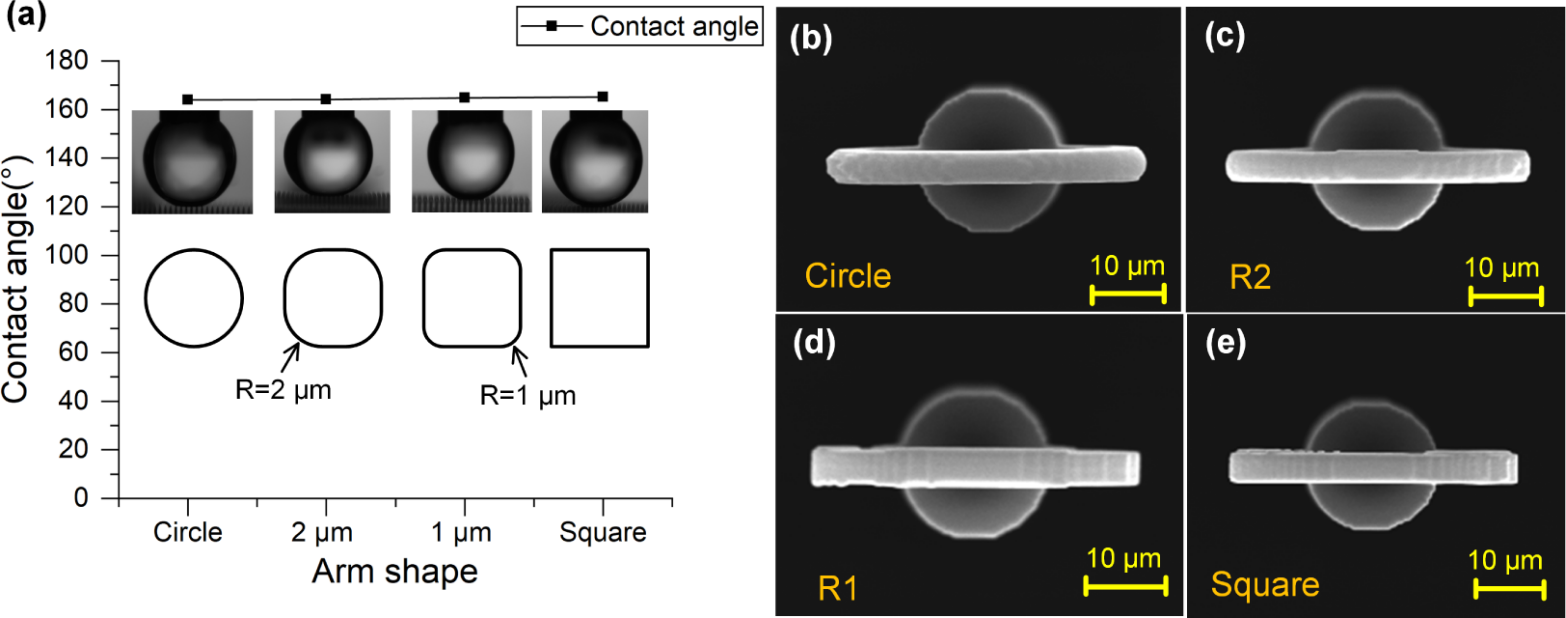
(a) Static contact angle as a function of arm cross-sectional shape. From left to right: circular, rounded square with fillet radius *R* = 2 μm, rounded square with *R* = 1 μm, and sharp-edged square. SEM images of Salvinia-inspired structures with circular (b), rounded square with fillet radius *R* = 2 μm (c), rounded square with *R* = 1 μm (d), and sharp-edged square­(e).

The degree of internal filling within the arms was examined to assess its effect on droplet behavior. As shown in Figure 7, no significant change in contact angle was observed when the ring-shaped arms transitioned from an open to a partially filled configuration. However, once the filling extended beyond half the radius—approaching a solid disk geometry—the structure transitioned to a conventional hydrophobic regime, with the CA reducing to 122.9°. Further filling had minimal additional effect. These results indicate that excessive internal mass within the arm negates the benefits of air retention and significantly alters the wetting regime.

**Figure 7:**
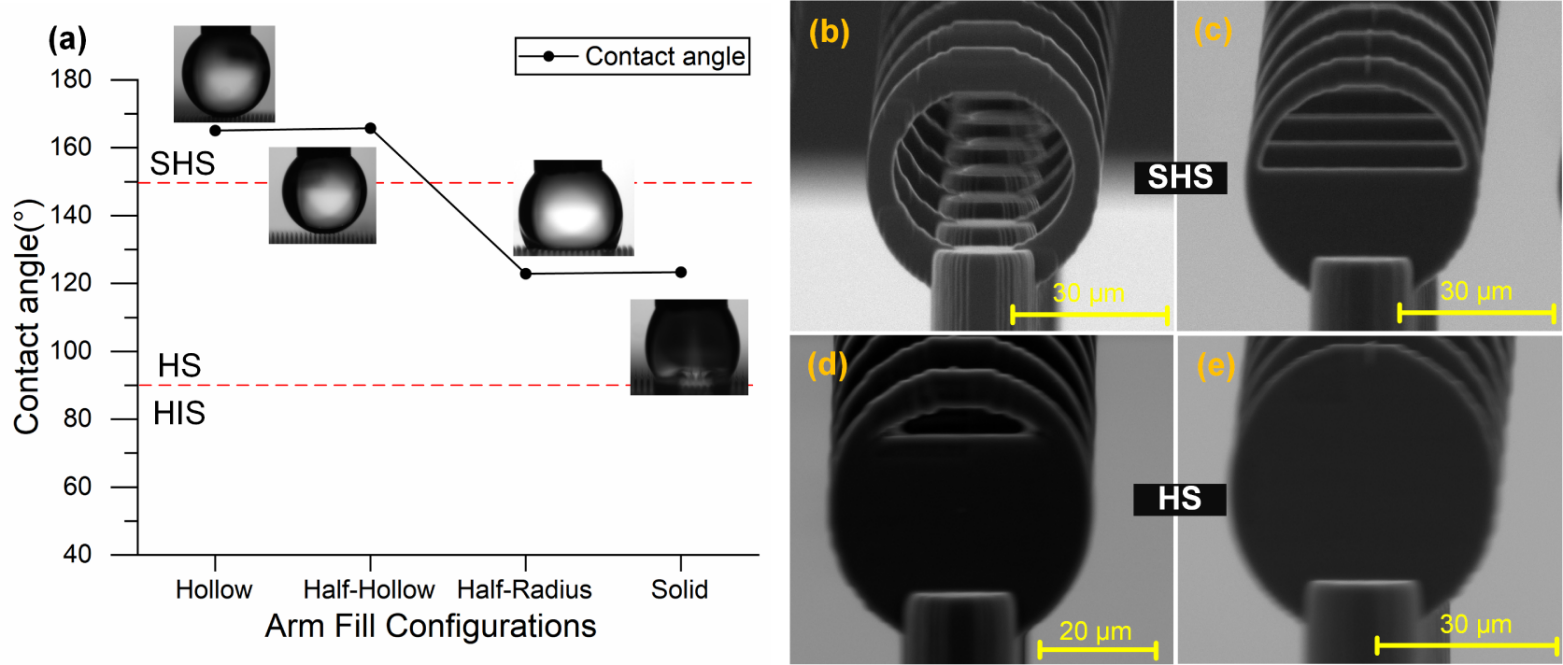
(a) Static contact angle as a function of different arm fill configurations: hollow, half-hollow, half-radius filled, and fully solid arms. (b–e) SEM images of representative structures corresponding to each fill condition: (b) hollow, (c) half-hollow, (d) half-radius, and (e) fully solid arms.

The influence of interstructure spacing on wettability was investigated next. Figure 8a shows how varying the spacing between structures from 40 to 90 μm affects the contact angle. A clear increase in CA was observed with increasing spacing, rising from 147.9° in the hydrophobic regime to 167.3°, entering the superhydrophobic domain. This behavior is likely related to enhanced droplet suspension and air retention between well-separated features. Figure 8b presents the relative deviation between designed and actual spacing values. Despite the high resolution of TPP, small discrepancies occur due to stage movement and material shrinkage during polymerization.^33–35^ Nonetheless, the maximum relative error remained within 1.4%, confirming the dimensional fidelity of the process.

**Figure 8:**
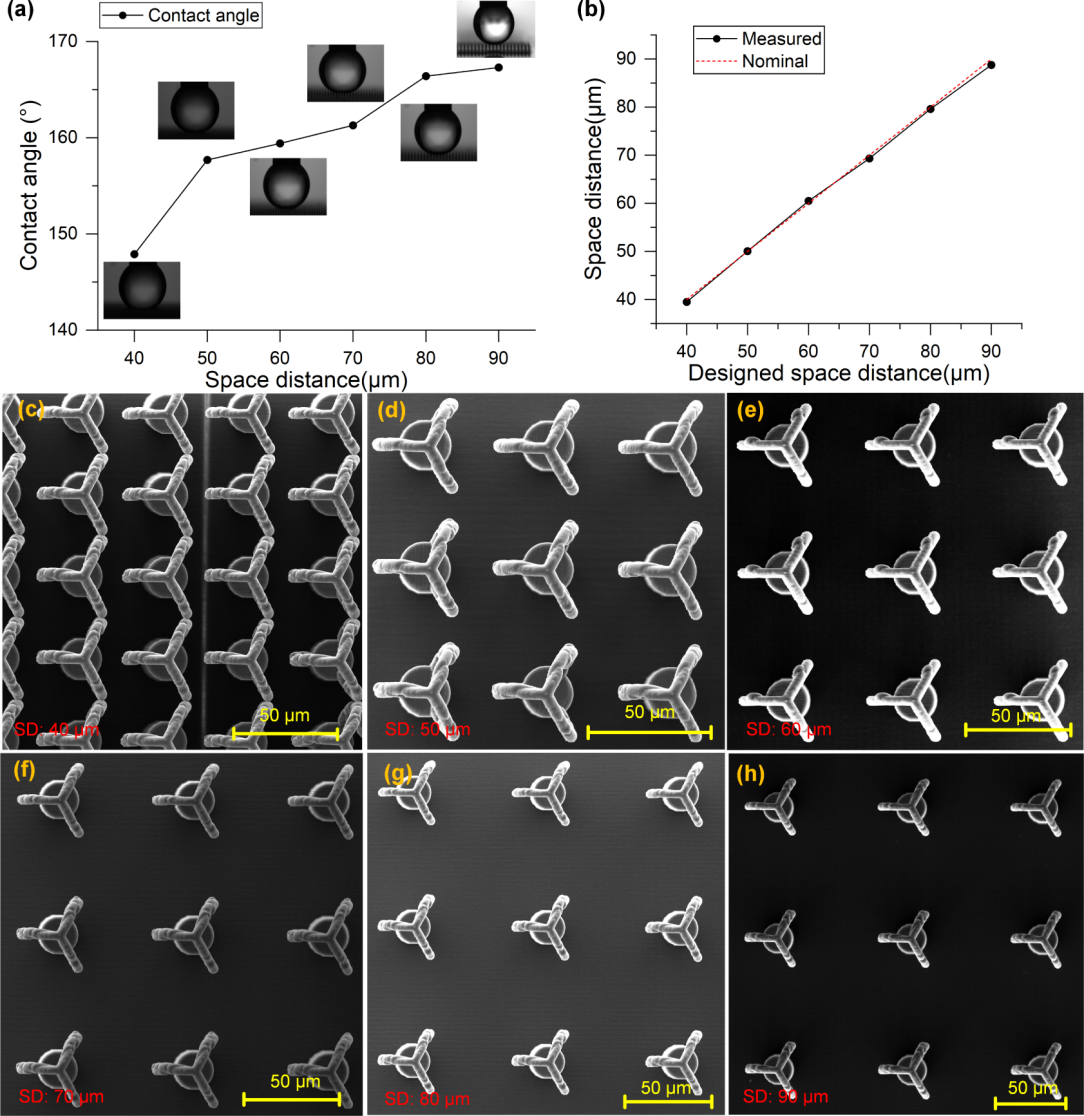
(a) Static contact angle as a function of surface spacing distance, (b) comparison between designed and measured space distances, (c–h) SEM images of Salvinia-inspired microstructures with increasing center-to-center distances (SD): 40 μm (c), 50 μm (d), 60 μm (e), 70 μm (f), 80 μm (g), and 90 μm (h).

In addition to lateral spacing, the effect of column height on contact angle was comprehensively evaluated under varying pitch conditions, as illustrated in Figure 9. SEM images in panels (a–c) show structures fabricated with heights of 0 μm, 60 μm, and 120 μm, enabling direct comparison of vertical confinement. Increasing the spacing distance led to a nonmonotonic trend in contact angle for each height condition, as reported in Figure 9d: the CA first increased slightly, plateaued, and then underwent a sharp drop, signaling the breakdown of the Cassie–Baxter regime and a transition to the Wenzel state.

**Figure 9:**
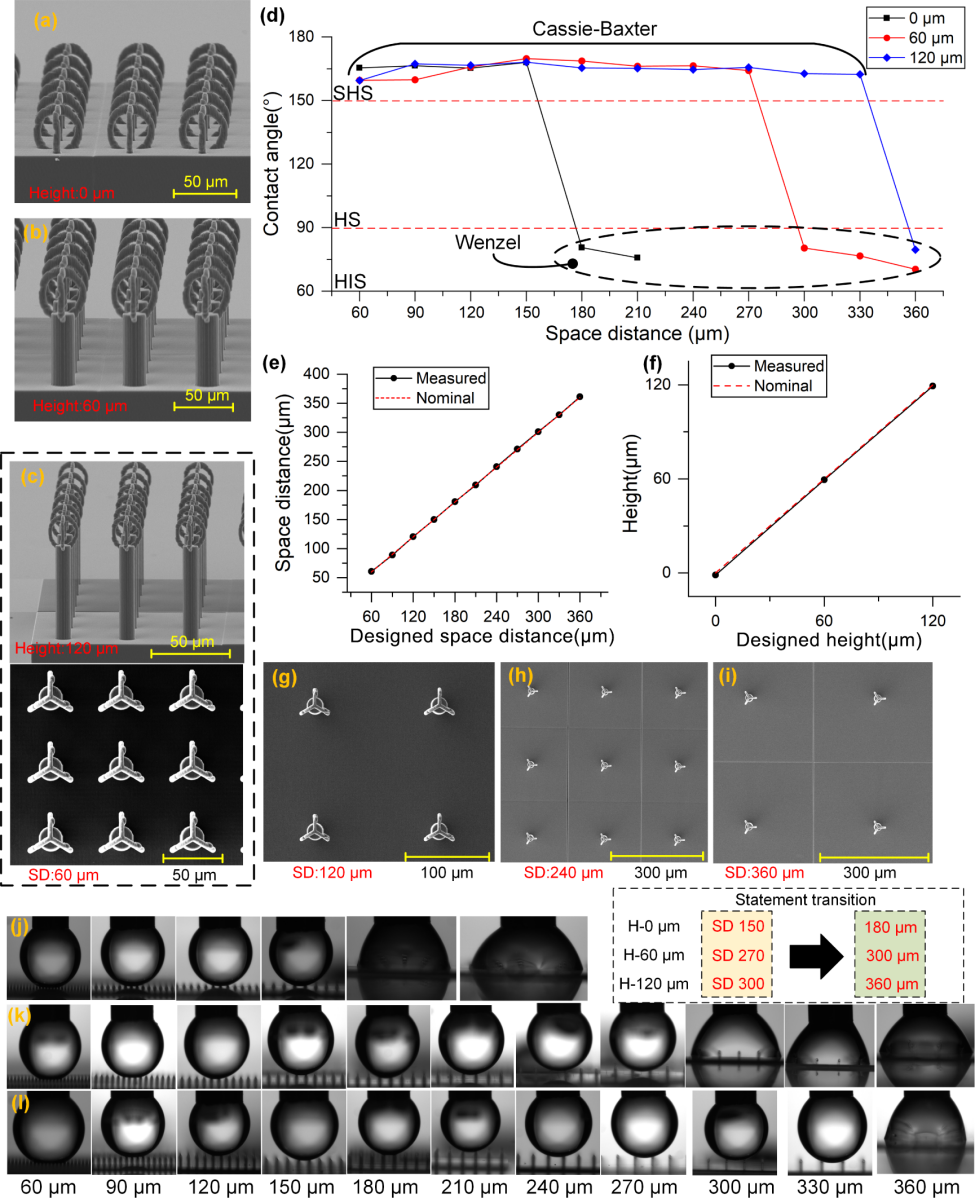
(a–c) SEM images of pillar-based structures with three different vertical heights: (a) 0 μm, (b) 60 μm, and (c) 120 μm; (d) static contact angle as a function of interstructure spacing for the three different heights; (e, f) comparison between designed and measured values for spacing (e) and height (f); (g–i) SEM top views of samples with increasing spacing distances: 120 μm, 240 μm, and 360 μm; (j–l) side-view droplet images for each height (0 μm, 60 μm, 120 μm) across various spacing distances.

This transition spacing was strongly dependent on column height. For flat structures (0 μm), the transition occurred between 150 and 180 μm; at 60 μm height, it shifted to 270 and 300 μm; and at 120 μm, it was delayed to 300 and 360 μm. These observations indicate that increased height contributes to greater droplet support and air retention, thereby extending the stability of the suspended wetting state over wider gaps. The highest contact angles observed at each height were: 166.4° (90 μm spacing, 0 μm height), 169.8° (150 μm spacing, 60 μm height), and 168.1° (150 μm spacing, 120 μm height). These results suggest that while height contributes to Cassie–Baxter stability, it does not necessarily guarantee a higher maximum contact angle compared to optimal spacing alone. Instead, height acts as a buffering factor, shifting the critical collapse point and broadening the design window for achieving superhydrophobic behavior.

Figure 9e,f confirms the fabrication precision for both spacing and height dimensions, with excellent agreement between nominal and measured values. SEM top views in panels (g–i) reveal the progressive loss of droplet support at increased spacing, consistent with the sudden drop in CA. Finally, side-view images in panels (j–l) show the visual transition in droplet morphology at each height condition, highlighting how the droplet begins to sag and spread significantly after the critical transition point is crossed.

### 3.2 Influence of Printing Parameters on Surface Wettability

Figure 10 presents the effects of printing parameters—specifically slicing distance (SLD) and hatching distance (HD)—on the wettability and surface morphology of 2-arm Salvinia-like structures.

**Figure 10:**
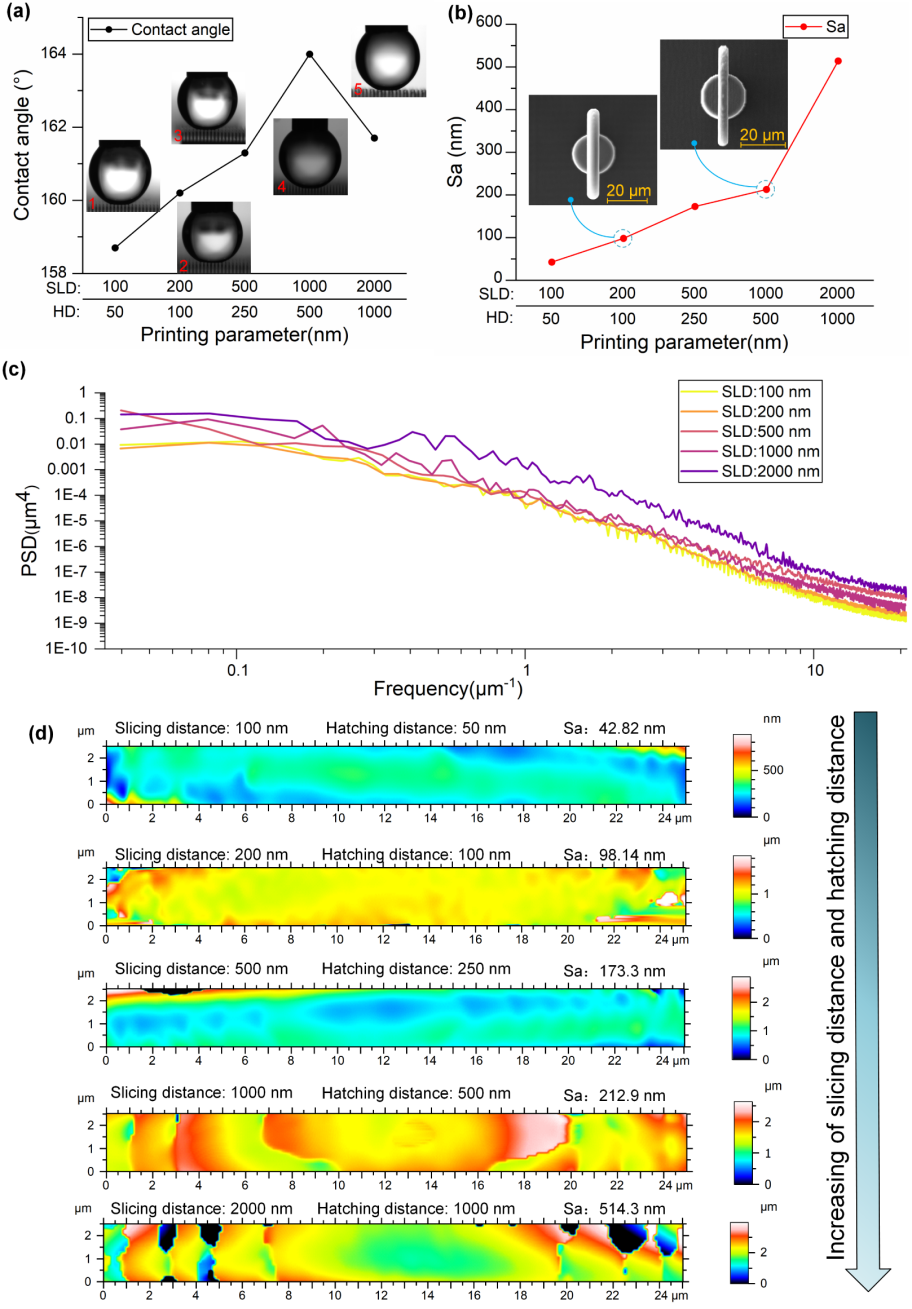
Influence of printing parameters—slicing distance (SLD) and hatching distance (HD)—on surface wettability and morphology of 2-arm Salvinia-like structures. (a) Static contact angle as a function of increasing SLD/HD combinations; (b) surface roughness (Sa); (c) power spectral density (PSD); (d) 3D surface topography maps (after shape removal).

These parameters directly influence voxel overlap and surface definition during two-photon polymerization (TPP). To avoid overexposure and associated bubble formation, the laser power was adjusted according to the selected SLD: 60%, 65%, and 80% were used for SLD values of 100, 200, and 500 nm, respectively.

As shown in Figure 10a, increasing SLD and HD from 100 nm/50 to 1000 nm/500 nm led to a gradual improvement in wettability, with the contact angle rising from 158.7° to a maximum of 164° at intermediate settings. This enhancement is likely due to increased surface roughness, which promotes air retention and supports Cassie–Baxter wetting. However, further increases in SLD and HD to 2000 nm/1000 nm resulted in a noticeable decline in contact angle to 161.7°, suggesting that excessive roughness or geometric distortion can compromise droplet suspension.

Figure 10b confirms this trend by correlating surface roughness (Sa) with printing resolution. As the printing parameters increased in scale, surface roughness rose markedly—from 42.82 nm at fine settings to 514 nm at the coarsest resolution. Insets show representative SEM images illustrating surface defects associated with poor voxel overlap or material shrinkage.

The power spectral density (PSD) curves in Figure 10c further support these findings. Finer slicing and hatching distances suppressed a broader range of high-frequency roughness components, resulting in smoother, more uniform surfaces. In contrast, larger SLD/HD values allowed more pronounced textural features to persist, increasing the macro-scale roughness.

Three-dimensional reconstructions of the structure cross sections (Figure 10d) illustrate the evolution of surface morphology in greater detail. As the SLD/HD increased, surface undulations became more prominent, and structural fidelity was progressively lost. This transition—from a smooth interface to irregular texturing—demonstrates the strong link between printing resolution, surface quality, and droplet behavior.

### 3.3 Advancing and Receding Contact Angles, Contact Angle Hysteresis, and Evaporation-Induced Transition

The wetting characteristics of the three structured surfaces were evaluated by measuring the advancing contact angle (ACA), receding contact angle (RCA), and contact angle hysteresis (CAH), as shown in Figure 11a. All samples exhibited high ACA values, confirming superhydrophobicity. N3-SD80-AD5 showed the highest ACA of 167.6°, followed by N2-SD60-AD5 (166.8°) and N6-SD60-AD2.5 (163.9°). More substantial variation was observed in RCA: N2 and N3 displayed high values of 143.6° and 135.5°, respectively, while N6 had a significantly lower RCA of 109.0°, resulting in a large contact angle hysteresis of 54.9°, compared to 23.2° (N2) and 32.1° (N3). Although all three samples exhibited high advancing contact angles (ACA > 150°), confirming their superhydrophobic nature, their large contact angle hysteresis (CAH > 20°) clearly places them into the category of high-adhesion superhydrophobic surfaces, also known as Salvinia-type surfaces, rather than low-adhesion lotus-effect surfaces. Salvinia-type surfaces are characterized by significant adhesion between water droplets and the surface due to their large CAH values, making droplets firmly adhere rather than easily roll off. Therefore, unlike typical lotus-effect surfaces known for effective self-cleaning due to minimal droplet adhesion (CAH typically below 5°), the tested samples here demonstrate limited self-cleaning capabilities. Nevertheless, such high-adhesion Salvinia-type structures may find suitable applications in fields such as water harvesting, controlled water evaporation, oil–water separation, stable air-layer recovery for underwater repellence, drag reduction, and thermal insulation, owing to their unique liquid adhesion and air retention properties.^36^

**Figure 11:**
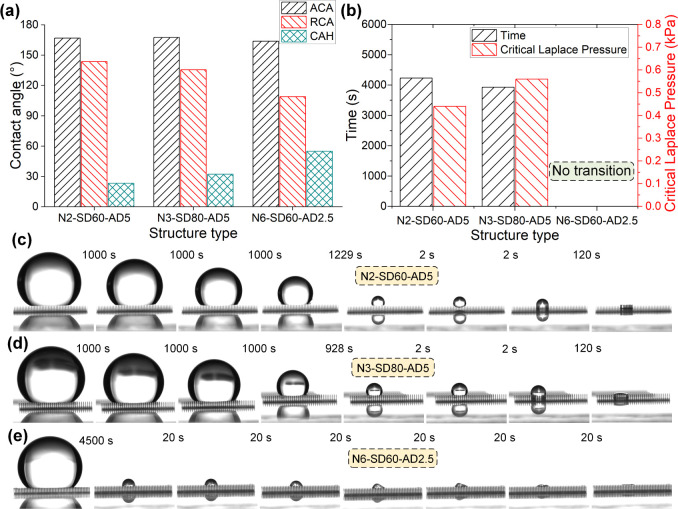
(a) Advancing contact angle (ACA), receding contact angle (RCA), and contact angle hysteresis (CAH) on three structured surfaces, (b) transition time and critical Laplace force on three structured surfaces, and side-view droplet evaporation on structured surfaces: (c) N2-SD60-AD5: droplet stays in Cassie–Baxter then collapses, (d) N3-SD80-AD5 droplet remains Cassie–Baxter then transitions to a partial Wenzel state. (e) N6-SD60-AD2.5 side-view: droplet stays in Cassie–Baxter with no transition was observed.

The Cassie-to-Wenzel transition behavior during droplet evaporation was investigated to assess the robustness of the composite interface (Figure 11b). N2-SD60-AD5 and N3-SD80-AD5 underwent transition after 4231 and 3930 s, with corresponding critical Laplace pressures of 0.44 and 0.56 kPa, indicating their finite resistance to liquid penetration. In contrast, N6-SD60-AD2.5 remained in the Cassie state throughout the entire observation period, and no transition was detected. Consequently, its transition time was recorded as 0 s in the data plot to indicate the absence of collapse during testing. Because no wetting transition occurred, a critical Laplace pressure could not be determined; however, this should be interpreted as evidence of superior resistance to wetting transition rather than the lack of measurable performance.

### 3.4 Fabrication Throughput and Efficiency

Figures 12 and 13 provide a comprehensive overview of how structural design and printing parameters influence the areal fabrication rate (AFR), a key metric for evaluating throughput in two-photon polymerization (TPP).

**Figure 12:**
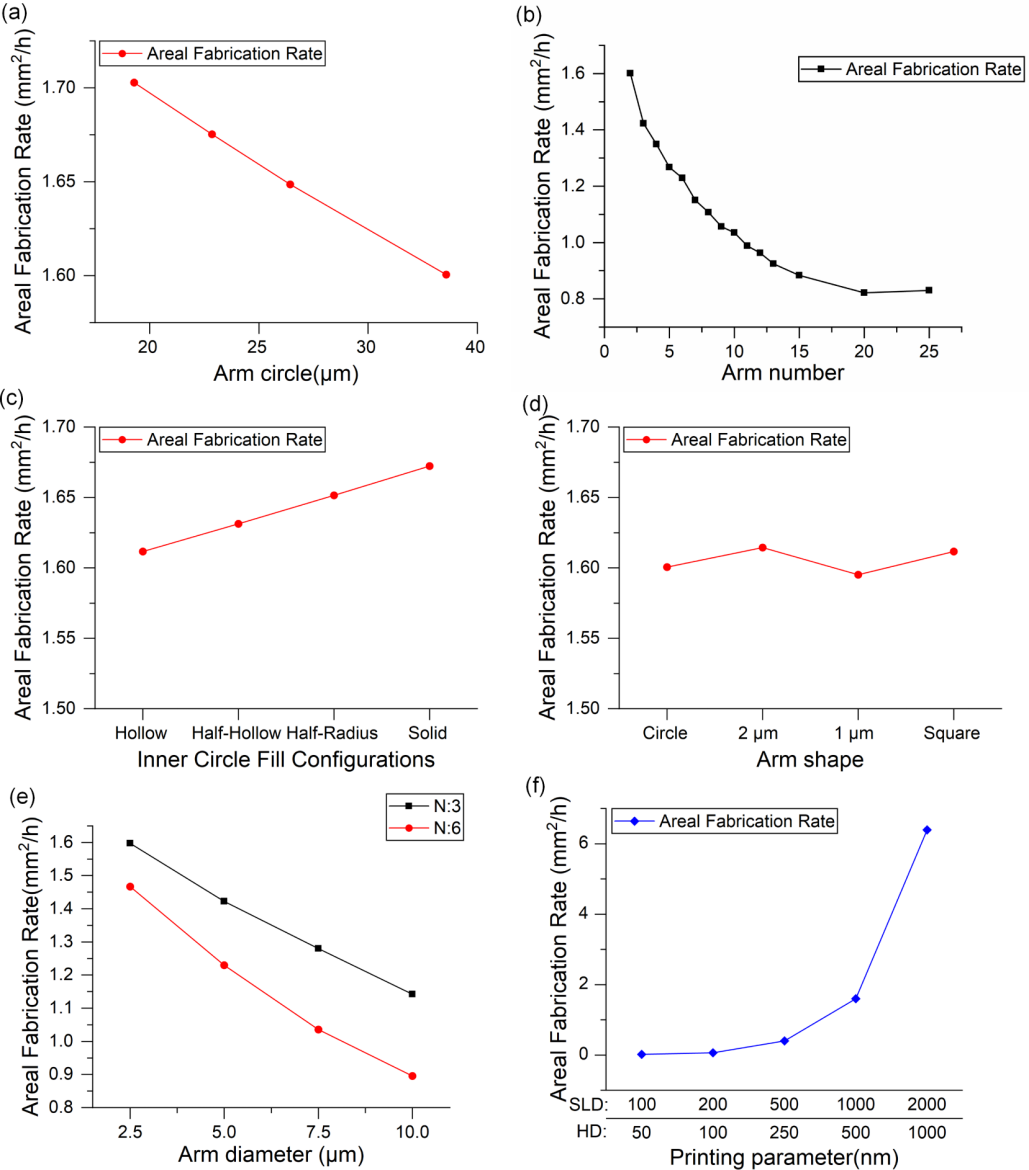
Areal fabrication rate (AFR) of Salvinia-like structures as a function of various geometric and printing parameters: (a) arm circle diameter; (b) arm number; (c) inner circle fill configurations; (d) arm shape; (e) arm diameter for different arm counts (*N* = 3 and *N* = 6); (f) slicing distance (SLD) and hatching distance (HD).

**Figure 13:**
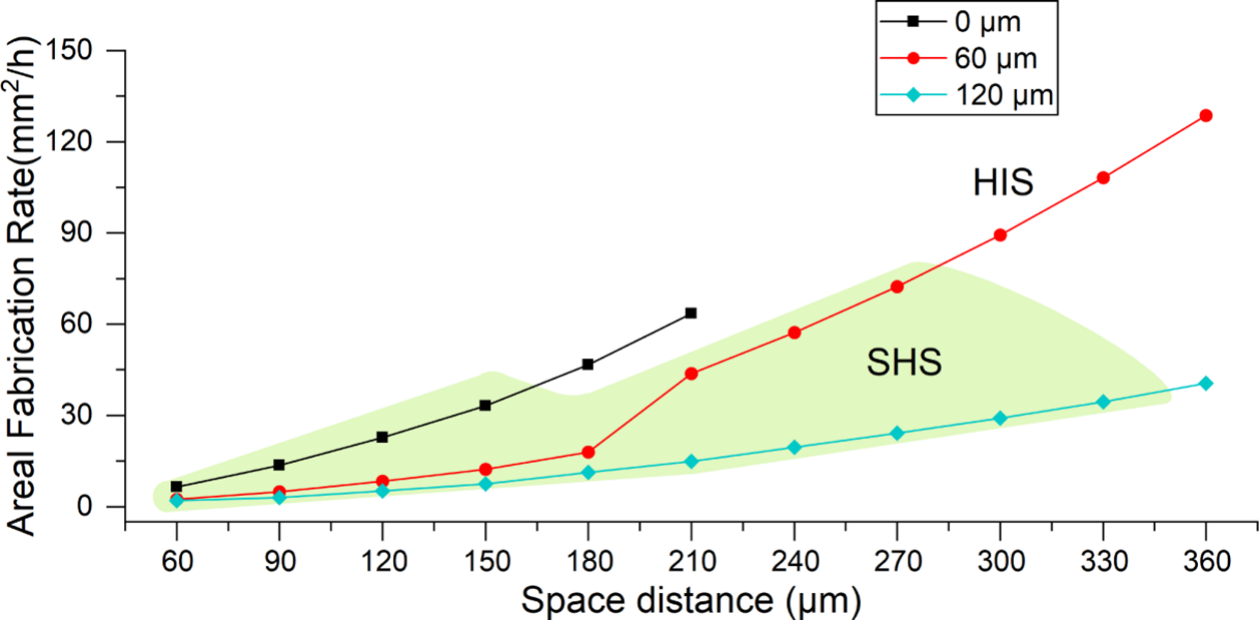
Areal fabrication rate (AFR) as a function of structure spacing for different pillar heights: (black) 0 μm, (red) 60 μm, and (blue) 120 μm; SHS and HIS regions are highlighted.

Figure 12a shows that as the arm circle diameter increases from 20 to 40 μm, AFR steadily decreases from 1.70 mm^2^/h to 1.60 mm^2^/h, reflecting the longer writing time required for larger features. In Figure 12b, increasing the number of arms from 2 to 25 results in a sharp drop in AFR from 1.60 mm^2^/h to 0.821 mm^2^/h, with a slight recovery to 0.830 mm^2^/h at the highest complexity. This suggests that increasing geometric intricacy significantly slows down fabrication, especially as arms multiply.

Figure 12c reports the influence of internal fill configurations. As the fill transitions from hollow to solid, the AFR shows a modest but consistent increase, from 1.611 mm^2^/h to 1.672 mm^2^/h. These results suggest that denser structures may allow more continuous printing paths, partially compensating for their volume.

In contrast, arm shape appears to have minimal impact on AFR, as shown in Figure 11d. The differences between circular, rounded, and square profiles are negligible, implying that cross-sectional geometry is not a limiting factor in printing speed under the tested conditions.

Figure 12e presents the combined effect of arm number and arm diameter. AFR declines significantly as the arm diameter increases from 2.5 to 10 μm. For three-arm configurations, it drops from 1.597 mm^2^/h to 1.142 mm^2^/h, while for six-arm structures, the decrease is more pronounced—from 1.467 mm^2^/h to 0.896 mm^2^/h. This confirms that both arm count and thickness directly affect fabrication time.

The most dramatic impact on AFR is associated with printing resolution. Figure 12f demonstrates that coarser slicing and hatching distances substantially accelerate fabrication. Increasing the slicing distance from 100 to 2000 nm and hatching from 50 to 1000 nm boosts AFR from 0.016 mm^2^/h to 6.391 mm^2^/h—highlighting a potential trade-off between resolution and production speed.

Figure 13 expands on this analysis by examining how spacing distance and structural height influence AFR. For a height of 0 μm, AFR increases steadily with spacing, from 6.525 mm^2^/h at 60 μm to 63.5 mm^2^/h at 210 μm. At 60 μm height, AFR improves even more markedly, peaking at 72.4 mm^2^/h at 270 μm. In contrast, for the tallest structures (120 μm), AFR rises more gradually, from 2.02 mm^2^/h to 34.47 mm^2^/h at 330 μm.

## 4 Discussions

### 4.1 Functional Design Principles for Superhydrophobicity

The primary aim of this work was to explore the potential of geometric design—enabled by two-photon polymerization (TPP)—to induce superhydrophobic behavior on intrinsically hydrophilic substrates without the aid of chemical modifications. The systematic design and fabrication of increasingly complex surface architectures established a clear correlation between micro/nanostructural layout and wetting performance. The results confirmed that appropriate topographic features alone could effectively stabilize the Cassie–Baxter regime and promote static contact angles well beyond 150°.

The structural design of Salvinia-inspired configurations proved particularly effective in achieving superhydrophobicity. Unlike conventional pillar arrays or solid 3D caps, branched geometries with distributed arm structures allowed for enhanced air entrapment and reduced liquid–solid contact area. Surfaces with two radially arranged arms and optimized ring diameters demonstrated the highest wetting resistance, reaching contact angles up to 164°. This demonstrates that roughness, morphological openness, edge curvature, and spatial symmetry are key in maintaining an air cushion beneath the droplet.

An important observation is that superhydrophobicity is not solely governed by the presence of microscale texture but also by the structure’s ability to trap and stabilize air at the solid–liquid interface. In this sense, the geometry acts as a functional scaffold for the Cassie–Baxter state, where the balance between capillary forces and topographic support dictates the overall wetting regime. Thus, geometric optimization becomes a critical pathway to bypass the need for low-surface-energy coatings in SHS design.

Furthermore, the study highlights the utility of TPP as a fabrication tool and as a means to isolate and analyze design parameters with submicron precision. By enabling the creation of consistent libraries of surface patterns, TPP provides a controlled framework for establishing quantitative relationships between design features and wetting behavior.

### 4.2 Interplay between Surface Architecture and Wetting Regime

The results demonstrate that surface architecture governs whether a structure can reach the superhydrophobic threshold and how stable this wetting state remains under varying geometrical constraints. Among all the design variables, the number of arms, their angular distribution, diameter, fill configuration, and spacing proved to have a collective and highly nonlinear influence on the wetting regime.

Reducing the ring diameter makes the structure more like a conventional 2D structure, whereas increasing the number of arms pushes it toward a normal 3D structure. Decreasing the ring diameter and adding more arms lowers the contact angle, indicating a threshold beyond which structural complexity compromises performance. This drop was particularly evident in odd-numbered configurations, which exhibited greater asymmetry and higher surface roughness than their even-numbered counterparts. The distinction was corroborated by both contact angle measurements and surface topography (Sa), suggesting that geometric disorder can hinder stable air retention.

Similarly, arm diameter played a critical role in modulating wettability. Thicker arms reduced the void fraction between structural elements, increasing the liquid–solid contact area and diminishing the Cassie–Baxter effect. Even though the global geometry was preserved, structures with six arms and 2.5 μm diameter achieved higher contact angles than their 10 μm counterparts. This underlines the importance of fine-scale features in shaping the local wetting interface.

The fill configuration of the arm’s interior showed an inflection point in the wetting transition. While hollow and half-filled structures maintained high contact angles, moving toward full fill introduced a collapse in air-layer continuity, causing the surface to revert to a hydrophobic state. This implies that external geometry and internal volume distribution must be carefully engineered to avoid disrupting the composite interface.

Another key observation is that superhydrophobicity emerged from a delicate balance between openness and support. Excessively narrow spacing can inhibit droplet suspension due to insufficient air cavity volume, while too wide a spacing promotes collapse into the Wenzel regime. Structural height further modulated this behavior, with taller geometries able to sustain the suspended state over larger pitches. The sharp wetting transition seen in spacing–height maps confirms that Cassie’s stability is not static but highly sensitive to spatial configuration.

Overall, the interplay between surface architecture and the wetting regime is driven by a multidimensional set of design parameters, in which small variations can produce significant shifts in performance.

The contact angle measurements reveal that while all selected three surfaces exhibit comparable advancing contact angles (ACAs) above 160°, their receding contact angles (RCAs) and contact angle hysteresis (CAH) differ significantly, reflecting distinct wetting dynamics during droplet retraction. N2-SD60-AD5 and N3-SD80-AD5, with relatively high RCA values and moderate CAH, suggest low contact line pinning and enhanced droplet mobility. In contrast, the low RCA and high CAH observed on N6-SD60-AD2.5 indicate strong pinning forces and high energy dissipation at the contact line, which can suppress droplet sliding despite the surface’s superhydrophobic nature. Interestingly, this strong pinning behavior did not lead to wetting transition during evaporation. On the contrary, N6-SD60-AD2.5 exhibited the most stable Cassie state, with no Cassie–Wenzel transition detected over the entire observation period. This contrasts with N2 and N3, which eventually transitioned into the Wenzel state despite their lower CAH. The inability to define a critical Laplace pressure for N6 does not indicate weak performance, but rather reflects the surface’s superior robustness against liquid impalement. These results suggest that while low CAH is often associated with improved antiwetting performance, strong contact line pinning—as seen in N6—can also contribute to enhanced Cassie state stability by inhibiting the kinetic pathways required for transition.

### 4.3 Morphological Control via Printing Parameters

While the structural design determines the theoretical wetting regime, the fidelity of the printed microfeatures strongly influences the actual surface behavior. This study demonstrated that printing parameters—particularly slicing distance (SLD) and hatching distance (HD)—directly affect the fabricated structures’ surface roughness, feature sharpness, and overall quality.

The experimental results showed that increasing SLD and HD clearly raises surface roughness (Sa), with values ranging from ∼43 nm at the finest resolution to over 500 nm at coarser settings. Corresponding changes in contact angle followed a nonmonotonic trend: moderate roughness enhanced superhydrophobicity, while excessive irregularity caused a decline. This is likely due to structural distortion, voxel overlap loss, and reduced air pocket definition.

Power spectral density (PSD) analysis further revealed that finer printing settings are more effective at suppressing high-frequency surface features, yielding smoother, more uniform textures. Coarser parameters, on the other hand, introduced greater microscale irregularities that disrupted droplet symmetry and potentially induced pinning.

The 3D topography maps and SEM imaging confirmed that surface fidelity is critical in determining whether the fabricated architecture matches its design intent. Poor fabrication resolution can undermine wetting performance even in geometries with optimized topology.

### 4.4 Trade-Off between Functional Performance and Fabrication Efficiency

A central challenge in SHS design is balancing wetting performance with fabrication throughput. The analysis of areal fabrication rate (AFR) across various geometries revealed a clear trade-off: as structural complexity increases, AFR decreases. More arms, greater diameters, and taller pillars all contribute to longer print times.

The data showed that low-complexity structures with fewer arms and smaller diameters offer higher AFRs, making them suitable for rapid prototyping or large-area fabrication. Conversely, highly optimized geometries—while functionally superior—require significantly more time to produce, with up to a 6–10× drop in throughput.

Interestingly, printing resolution was the most decisive factor in AFR variation. Increasing SLD/HD from 100/50 nm to 2000/1000 nm raised AFR by nearly 2 orders of magnitude. However, this gain came at the expense of surface fidelity and contact angle performance, confirming the inverse relationship between quality and speed.

The results also indicate that spacing and height must be co-optimized. For example, AFR increased steadily with spacing at all heights, but the highest throughput gains were recorded at intermediate pillar heights (∼60 μm), where printing speed and functional wetting behavior intersected most favorably.

### 4.5 Design Guidelines for Scalable SHS Fabrication

Based on the comprehensive parametric study, several guidelines emerge for designing superhydrophobic surfaces that balance performance and production efficiency:

- Favor symmetric arm arrangements (even-numbered) and moderate diameters (∼25–30 μm) to maximize contact angle while minimizing roughness-induced pinning.
- Use 2 arm configurations for optimal balance between droplet suspension and fabrication time.
- Apply partial fill to arms (∼50%) to maintain high contact angles without significantly increasing material volume or print duration.
- In functional designs, limit arm diameter and global feature complexity to preserve AFR above 1 mm^2^/h.
- Maintain pillar height between 60–120 μm and spacing below the collapse threshold to ensure Cassie state retention.
- Select intermediate slicing/hatching settings (e.g., 1000/500 nm) to balance structural fidelity with productivity.

## 5 Conclusions

This study demonstrates the potential of two-photon polymerization (TPP) as a high-resolution, maskless fabrication technique to achieve superhydrophobicity on intrinsically hydrophilic surfaces solely through structural design. This work provides new insights into the design-function relationships that govern surface-wetting behavior by engineering a comprehensive library of bioinspired micro/nanostructures and performing detailed wettability and morphological characterizations.

The experimental findings and design analysis lead to the following key conclusions:

- Salvinia-inspired geometries enable a reliable transition from hydrophilic to superhydrophobic regimes without the need for chemical surface modifications.
- The superhydrophobic state results from a complex interplay between architectural parameters, including arm circle diameter, number of arms, arm thickness, fill configuration, pillar height, and spacing distance. These parameters collectively modulate the liquid–solid contact area and the capacity to trap air.
- Increasing the number of arms beyond a threshold leads to a gradual decline in contact angle, especially in asymmetrical configurations. Even-numbered structures generally have smoother morphology and higher contact angles than their odd-numbered counterparts.
- Structural fidelity and surface roughness, governed by TPP parameters such as slicing and hatching distances, play a pivotal role in maintaining wetting performance. Moderate roughness supports air entrapment, while excessive roughness disrupts the Cassie–Baxter regime.
- Fabrication efficiency, expressed as areal fabrication rate (AFR), increases with simpler geometries, wider spacing, reduced height, and coarser printing parameters. However, these gains must be balanced against the structural requirements for sustaining superhydrophobicity.
- This work presents a versatile design strategy for fabricating scalable, functionally robust superhydrophobic surfaces through geometry alone. The results lay the foundation for future applications in microfluidics, antifouling coatings, and biomimetic surface engineering, where chemical-free wettability control is desirable.

## Data Availability

Data will be made available on request.

## References

[1] Dalawai S. P., Aly M. A. S., Latthe S. S., Xing R., Sutar R. S., Nagappan S., Ha C., Sadasivuni K. K., Liu S. (2020). Recent Advances in Durability of Superhydrophobic Self-Cleaning Technology:
A Critical Review. Prog. Org. Coat..

[2] Yang Y., Li X., Zheng X., Chen Z., Zhou Q., Chen Y. (2018). 3D-Printed
Biomimetic Super-Hydrophobic Structure for Microdroplet Manipulation
and Oil/Water Separation. Adv. Mater..

[3] Park S., Sung J., So H. (2022). Three-Dimensional Printing-Assisted
All-in-One Surfaces Inspired by Peristome Structures for Water–Oil
Separation. Surfaces Interfaces.

[4] Huang J., Wang Q., Wu Z., Ma Z., Yan C., Shi Y., Su B. (2021). 3D-Printed Underwater
Super-Oleophobic Shark Skin toward
the Electricity Generation through Low-Adhesion Sliding of Magnetic
Nanofluid Droplets. Adv. Funct. Mater..

[5] Deng W., Su Y., Zhang C., Wang W., Xu L., Liu P., Wang J., Yu X., Zhang Y. (2023). Transparent Superhydrophilic
Composite Coating with Anti-Fogging and Self-Cleaning Properties. J. Colloid Interface Sci..

[6] Xue C. H., Guo X. J., Ma J. Z., Jia S. T. (2015). Fabrication of Robust
and Antifouling Superhydrophobic Surfaces via Surface-Initiated Atom
Transfer Radical Polymerization. ACS Appl. Mater.
Interfaces.

[7] Guo Z., Liu W. (2007). Biomimic from the Superhydrophobic Plant Leaves in Nature: Binary
Structure and Unitary Structure. Plant Sci..

[8] Das S., Kumar S., Samal S. K., Mohanty S., Nayak S. K. (2018). A Review
on Superhydrophobic Polymer Nanocoatings: Recent Development and Applications. Ind. Eng. Chem. Res..

[9] Cohen N., Dotan A., Dodiuk H., Kenig S. (2016). Superhydrophobic Coatings
and Their Durability. Mater. Manuf. Process..

[10] Bayer I. S. (2020). Superhydrophobic
Coatings from Ecofriendly Materials and Processes: A Review. Adv. Mater. Interfaces.

[11] Mortazavi V., Khonsari M. M. (2017). On the Degradation of Superhydrophobic Surfaces: A
Review. Wear.

[12] Mackay D., Powell D. E., Woodburn K. B. (2015). Bioconcentration
and Aquatic Toxicity
of Superhydrophobic Chemicals: A Modeling Case Study of Cyclic Volatile
Methyl Siloxanes. Environ. Sci. Technol..

[13] Jafari R., Cloutier C., Allahdini A., Momen G. (2019). Recent Progress and
Challenges with 3D Printing of Patterned Hydrophobic and Superhydrophobic
Surfaces. Int. J. Adv. Manuf. Technol..

[14] Gaxiola-López J. C. (2022). 3D
printed parahydrophobic surfaces as multireaction platforms. Langmuir.

[15] Sung J., Lee H. M., Yoon G. H., Bae S., So H. (2023). One-Step Fabrication
of Superhydrophobic Surfaces with Wettability Gradient Using Three-Dimensional
Printing. Int. J. Precis. Eng. Manuf. Green
Technol..

[16] Bruzzone A. A. G., Costa H. L., Lonardo P. M., Lucca D. A. (2008). Advances in Engineered
Surfaces for Functional Performance. CIRP Ann..

[17] Zhang S., Li S., Wan X., Ma J., Li N., Li J., Yin Q. U. (2021). High-Resolution and Large-Size Three-Dimensional Structure
Manufacturing through High-Efficiency Two-Photon Polymerization Initiators. Addit. Manuf..

[18] Xiang Y., Huang S., Huang T. Y., Dong A., Cao D., Li H., Xue Y., Lv P., Duan H. (2020). Superrepellency of
Underwater Hierarchical Structures on Salvinia Leaf. Proc. Natl. Acad. Sci. U. S. A..

[19] Liimatainen V., Drotlef D. M., Son D., Sitti M. (2020). Liquid-Superrepellent
Bioinspired Fibrillar Adhesives. Adv. Mater..

[20] Lin Y., Zhou R., Xu J. (2018). Superhydrophobic Surfaces Based on
Fractal and Hierarchical Microstructures Using Two-Photon Polymerization:
Toward Flexible Superhydrophobic Films. Adv.
Mater. Interfaces.

[21] Berwind M. F., Hashibon A., Fromm A., Gurr M., Burmeister F., Eberl C. (2017). Rapidly Prototyping
Biocompatible Surfaces with Designed Wetting
Properties via Photolithography and Plasma Polymerization. Microfluid. Nanofluid.

[22] Lantada A. D., Hengsbach S., Bade K. (2017). Lotus-on-Chip: Computer-Aided Design
and 3D Direct Laser Writing of Bioinspired Surfaces for Controlling
the Wettability of Materials and Devices. Bioinspir.
Biomim..

[23] Tricinci O., Terencio T., Mazzolai B., Pugno N. M., Greco F., Mattoli V. (2015). 3D Micropatterned Surface Inspired by Salvinia molesta
via Direct Laser Lithography. ACS Appl. Mater.
Interfaces.

[24] Tricinci O., Pignatelli F., Mattoli V. (2023). 3D Micropatterned Functional Surface
Inspired by Salvinia molesta via Direct Laser Lithography for Air
Retention and Drag Reduction. Adv. Funct. Mater..

[25] Wenzel R. N. (1936). Resistance
of Solid Surfaces to Wetting by Water. Ind.
Eng. Chem..

[26] Cassie A. B. D., Baxter S. (1944). Wettability of Porous Surfaces. Trans. Faraday Soc..

[27] Bico J., Thiele U., Quéré D. (2002). Wetting of Textured Surfaces. Colloids Surf., A.

[28] Barthlott W., Schimmel T., Wiersch S., Koch K., Brede M., Barczewski M., Walheim S., Weis A., Kaltenmaier A., Leder A., Bohn H. F. (2010). The Salvinia Paradox: Superhydrophobic
Surfaces with Hydrophilic Pins for Air Retention under Water. Adv. Mater..

[29] Maruo S., Nakamura O., Kawata S. (1997). Three-Dimensional
Microfabrication
with Two-Photon-Absorbed Photopolymerization. Opt. Lett..

[30] LaFratta C. N., Fourkas J. T., Baldacchini T., Farrer R. A. (2007). Multiphoton Fabrication.
Angew. Angew. Chem., Int. Ed..

[31] Law K. Y. (2014). Definitions
for Hydrophilicity, Hydrophobicity, and Superhydrophobicity: Getting
the Basics Right. J. Phys. Chem. Lett..

[32] Huhtamäki T., Tian X., Korhonen J. T., Ras R. H. (2018). Surface-Wetting
Characterization Using Contact-Angle Measurements. Nat. Protoc..

[33] Zhou X., Hou Y., Lin J. (2015). A Review on
the Processing Accuracy of Two-Photon Polymerization. AIP Adv..

[34] Denning R. G., Blanford C. F., Urban H., Bharaj H., Sharp D. N., Turberfield A. J. (2011). The Control
of Shrinkage and Thermal Instability in
SU-8 Photoresists for Holographic Lithography. Adv. Funct. Mater..

[35] Sun Q., Ueno K., Misawa H. (2012). In Situ Investigation
of the Shrinkage
of Photopolymerized Micro/Nanostructures: The Effect of the Drying
Process. Opt. Lett..

[36] Bing W., Wang H., Tian L., Zhao J., Jin H., Du W., Ren L. S. S. (2021). Small
Structure, Large Effect: Functional Surfaces
Inspired by Salvinia Leaves. Small Struct..

